# Effects of Age and Biological Age-Determining Factors on Telomere Length in Type 2 Diabetes Mellitus Patients

**DOI:** 10.3390/medicina60050698

**Published:** 2024-04-24

**Authors:** Jawaria Ali Tariq, KaleemUllah Mandokhail, Naheed Sajjad, Abrar Hussain, Humera Javaid, Aamir Rasool, Hummaira Sadaf, Sadia Javaid, Abdul Rauf Durrani

**Affiliations:** 1 Department of Biotechnology, Sardar Bahadur Khan Women’s University, Quetta 87300, Pakistan; jawaria_ali_tariq@yahoo.com (J.A.T.); drnaheedsajjad@gmail.com (N.S.); chokohollikhhf@gmail.com (H.J.); hummairas1@gmail.com (H.S.); rorquel_roller@yahoo.com (S.J.); 2 Department of Microbiology, University of Balochistan, Quetta 87300, Pakistan; drkaleemullah@gmail.com; 3 Department of Biotechnology, Balochistan University of Information Technology, Engineering and Management Sciences, Quetta 87300, Pakistan; 4 Institute of Biochemistry, University of Balochistan, Quetta 87300, Pakistan; rasool.amir@gmail.com; 5 Maria Sklodowska-Curie National Research Institute of Oncology, Silesian University of Technology, 02-781 Warsaw, Poland; 6 Provincial Reference Laboratory (PRL), Fatima Jinnah General and Chest Hospital, Quetta 87300, Pakistan; abdulraufdurrani@gmail.com

**Keywords:** telomere length, T/S ratio, diabetes mellitus type 2, molecular aging, qPCR assay

## Abstract

*Background and Objectives:* Telomere length (TL) undergoes attrition over time, indicating the process of aging, and is linked to a higher risk of diabetes mellitus type 2 (DM-2). This molecular epidemiological study investigated the correlation between leukocyte TL variations and determinants of molecular aging in 121 Pakistani DM-2 patients. *Materials and Methods:* The ratio of telomere repeats to the SCG copy number was calculated to estimate the TL in each sample through qPCR assays. *Results:* In this study, smaller mean TLs were observed in 48.8% of males (6.35 ± 0.82 kb), 3.3% of underweight patients (5.77 ± 1.14 kb), 61.2% of patients on regular medication (6.50 ± 0.79 kb), 9.1% with very high stress levels (5.94 ± 0.99 kb), 31.4% of smokers (5.83 ± 0.73 kb), 40.5% of patients with low physical activity (6.47 ± 0.69 kb), 47.9% of hypertensive patients (5.93 ± 0.64 kb), 10.7% of patients with DM-2 for more than 15 years, and 3.3% of patients with a delayed onset of DM-2 (6.00 ± 0.93 kb). *Conclusion:* This research indicated a significant negative correlation (R^2^ = 0.143) between TL and the age of DM-2 patients. This study demonstrated that the correlation of telomere length with age in DM-2 patients was also influenced by various age-determining factors, including hypertension and smoking habits, with significant strong (R^2^ = 0.526) and moderate (R^2^ = 0.299) correlations, respectively; sex, obesity, the stress level and age at the onset of diabetes with significant weak correlations (R^2^ = 0.043, 0.041, 0.037, and 0.065, respectively), and no significant correlations of medication routine, rate of physical activity, and the durations of DM-2 with age-adjusted telomere length. These results challenge TL as the sole marker of aging, thus highlighting the need for further research to understand underlying factors and mitigate the effect of aging or premature aging on diabetic patients.

## 1. Introduction

Chronic hyperglycemia indicates type 2 diabetes mellitus (DM-2), a heterogeneous illness manifested by altered insulin production and insulin resistance [1]. DM-2 accounts for >90% of all cases of diabetes mellitus [2]. In addition to the elderly and middle-aged, young people are becoming more and more affected by this condition, especially in non-Caucasian communities [3].

Genetic susceptibility in different populations and the combination of several environmental variables lead to type 2 diabetes mellitus [4]. Diabetes is one of the major health concerns worldwide, especially in Asian countries. Based on recent studies, one in four individuals in the general population in Pakistan has DM-2 [5,6]. Pakistan is a developing nation with a high prevalence of diabetes, as shown by a recent survey through which it was found that 26% of adults in the general population had the disease [5].

Telomeres (DNA and histone protein complexes) shield the chromosome ends from fusion and destruction [7]. Telomere length (TL) undergoes attrition over time, indicating the process (and a potential cause) of aging in human tissues [8]. According to many studies of humans, a short TL assessed in leukocytes is linked to a higher risk of age-related conditions, such as type 2 diabetes [9] and cardiovascular disease [10], along with the individual’s lifespan and mortality rate [11]. Leukocyte TL is also linked to environmental exposures (e.g., radiation, smoking cigarettes), health variables (e.g., cholesterol, obesity), and lifestyle factors (e.g., physical activity, dietary habits) [12,13].

Several mechanisms lead to the accelerated molecular aging process in diabetic patients, including telomere shortening, accumulation of advanced glycation end products (AGEs), cellular senescence, inflammation, oxidative stress, and epigenetic modifications. These molecular aging mechanisms can lead to many other age-related disorders, along with diabetes [14,15].

Different studies have explained the effect of diabetes on telomere shortening, some of which suggested an association between diabetes and accelerated aging at the molecular level [15,16]. At the same time, few studies have reported no significant correlation between telomere length and the age of DM-2 patients [17]. A constant rise in the prevalence of DM-2 in Asian countries emphasizes the need for molecular and clinical investigations of this disease, particularly in the elderly population. As no epidemiological data related to Pakistan associating telomere length with the chronological age of DM-2 patients is available to date, the scope of this molecular epidemiological study is to find the telomere length dynamics in leukocytes from diabetic patients and to correlate age-adjusted telomere length variations with various determinants of the molecular aging process in Pakistani patients, with a future perspective to identify biological variables leading to premature molecular aging in DM-2 patients.

## 2. Materials and Methods

### 2.1. Experimental Design

The experimental design was planned as suggested by Cawthon [18] and Axelrad et al. [19] for determining telomere lengths by quantitative PCR. Cell populations from different tissues may have different replicative histories, along with the telomere length in those cells. For this research, the telomere length of the leukocytes was measured. For each DNA sample from diabetic patients, the ratio of telomere repeat copy number (T) to SCG copy numbers (S) was calculated to estimate telomere length. The T/S ratio and telomere length are directly proportional because the primer–DNA binding tendency (during initial PCR cycles) and telomere length are directly associated. Thus, both copy numbers (T and S) were measured by comparing the difference in cycle threshold (Ct value) of samples with primers for telomeres and the single copy gene (SCG).

### 2.2. Inclusion Criteria

Individuals above the age of 39 years with a medical history of type 2 diabetes mellitus were selected for this study. A detailed scrutiny of 350 DM-2 patients was performed through a questionnaire-based survey to minimize the effect of extraneous variables, and 121 patients were selected as a homogenized cohort from the population based on the following:

- Geographical characteristics (i.e., samples from areas/societies of city away from industrial pollution, and samples from developed residential areas free from a congested population and heavy traffic, also samples from migrants or new residents were avoided);
- The patient’s medical history (patients having a medical history of a cardiac or any metabolic disorder, any physical disability, genetic disorder, cancer, or any other chronic disease were skipped);
- The use of supplements (no samples were collected from the patients taking vitamins or supplements through pills or injections);
- Sleep time (samples were collected only from patients with 7–8 h of sleep per day);
- Extra physical activity (patients performing excessive exercise and gym workouts were not considered in this study);
- Tobacco consumption other than smoking (patients taking smokeless or chewable forms of tobacco were not considered suitable candidates for sampling);
- Lifestyle variations (individuals with exhaustive working hours of >6 h/day at the job, individuals below the poverty line, individuals with luxurious lifestyles, individuals with high consumption of fats in diet were also not considered; moreover, patients taking medicines other than metformin were also excluded from this study);
- Marital status (only married individuals were considered for further analysis);
- Fertility (infertile, menopausal, and post-menopausal patients were not analyzed further).

### 2.3. Sample Collection

Fresh blood was collected from selected 121 diabetic patients (>39 years of age) voluntarily through the standard venipuncture technique in BD sterile vacutainer blood tubes with EDTA (Becton Dickinson UK Ltd., Oxford, UK; Cat. No. 366643). All the samples were processed for the DNA extraction protocol on the same day of sample collection to maintain uniformity in the procedure.

### 2.4. DNA Extraction

For the optimization of the DNA extraction protocol and to maintain the integrity and purity of isolated DNA molecules, different methods were scrutinized. The organic method by Shen [20] using a phenol–chloroform solution was found to extract intact and pure DNA molecules, showing a sharp bulky band with no smearing during gel electrophoresis. Therefore, all the blood samples were processed using the organic method for DNA extraction. Extracted DNA was stored in low Tris–EDTA buffer (TE^−4^, pH = 7.5) at −20 degrees Celsius until further use (not more than three days). Before amplification, the quantity and quality of DNA were measured using a NanoDrop spectrophotometer (Thermo Scientific^TM^, Waltham, MA USA; Cat. No. ND-2000) by the A260/280 ratio. * All procedures were performed in a biological safety cabinet.

### 2.5. Oligomers

All oligomers, including standards and primers, were diluted in PCR-grade water to make a stock concentration of 100 pmoles/μL and then kept at −20 °C until further required. Working stocks (10 pmoles/μL) of all the primers were freshly prepared before starting the reaction, and the remaining working primers were stored at 4 °C (not longer than two weeks). Standards and primers (Supplementary Table S1) for the SCG (β-Globin) and telomeres were used, as stated by O‘Callaghan and Fenech [21].

### 2.6. Serial Dilutions

Oligomer standards (both telomeric and SCG standards) were serially diluted in PCR-grade water by the dilution factor (1.68) suggested by O‘Callaghan and Fenech [21] to generate the standard curve of Ct values for assay, as shown in Table 1.

Dilutions of the standard oligomers were then added to the PCR tubes with ultra-clear caps for qPCR assays. Additionally, 20 ng of plasmid DNA (pBR-322) was added to each tube of serially diluted standards to maintain the overall mass of the DNA molecule.

### 2.7. Normalization

Varying DNA concentrations may give different average telomere lengths in each sample. To avoid this problem and pipetting errors, DNA normalization was performed by maintaining a consistent concentration of DNA (5 ng/μL) in each sample and diluting extracted DNA samples with PCR-grade water, as practiced by Axelrad et al. [19].

### 2.8. qPCR Protocol

After diluting all the DNA samples to a final concentration of 5 ng/μL, two master mixes of PCR reagents were prepared: one with telomere forward and reverse primers and the other with the primer pair for the SCG. Aliquots were prepared for no template control and standards plus an extra 5% for pipetting errors (Supplementary Table S2).

All samples were run on a SaCycler-96 (Sacace Biotechnology, Como, Italy) with the SaCycler-96 Real-Time PCR V.7.3 (Sacace Biotechnology, Como, Italy). Both telomere and SCG reactions were run separately using the following program. The reaction program was set as follows: initial denaturation of 10 min followed by 35 cycles of 95 degrees Celsius for 15 s, 60 degrees Celsius for 60 s, and finally a dissociation (or melting) curve.

### 2.9. Data Analysis

Amplification was observed in standards and samples according to the procedure performed by O‘Callaghan and Fenech [21]. The baseline was set (because standards were amplified earlier than the samples), and a standard curve was generated using the C_t_ readings.

**Figure 1:**
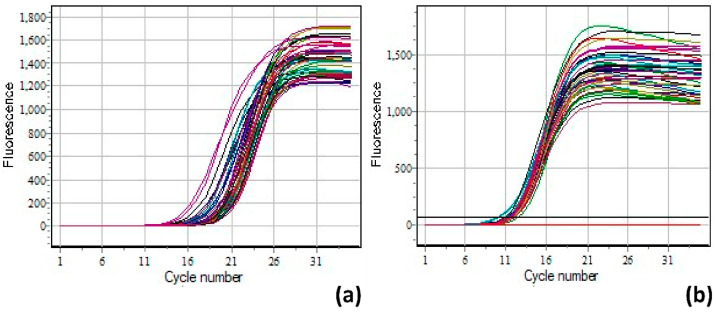
The qPCR assay (colored lines represent each sample). (**a**) Amplification curve of telomeric regions and (**b**) amplification curve of the SCG to evaluate the cycle threshold.

Both telomere and SCG assays were run on each sample. The number of cycles required for fluorescence detection to attain the exponential curve (Ct value) varies between these two assays for a single sample due to the varying DNA copy numbers generated in each assay (Figure 1).

A mean TL of 4270 bp in leukocytes is equal to one T/S ratio unit (qPCR cycles of the telomere standard run over the cycles of the SCG standard run) [22]. In this study, a ratio of 0.98 corresponds to an average of 4185 bp.

**Figure 2:**
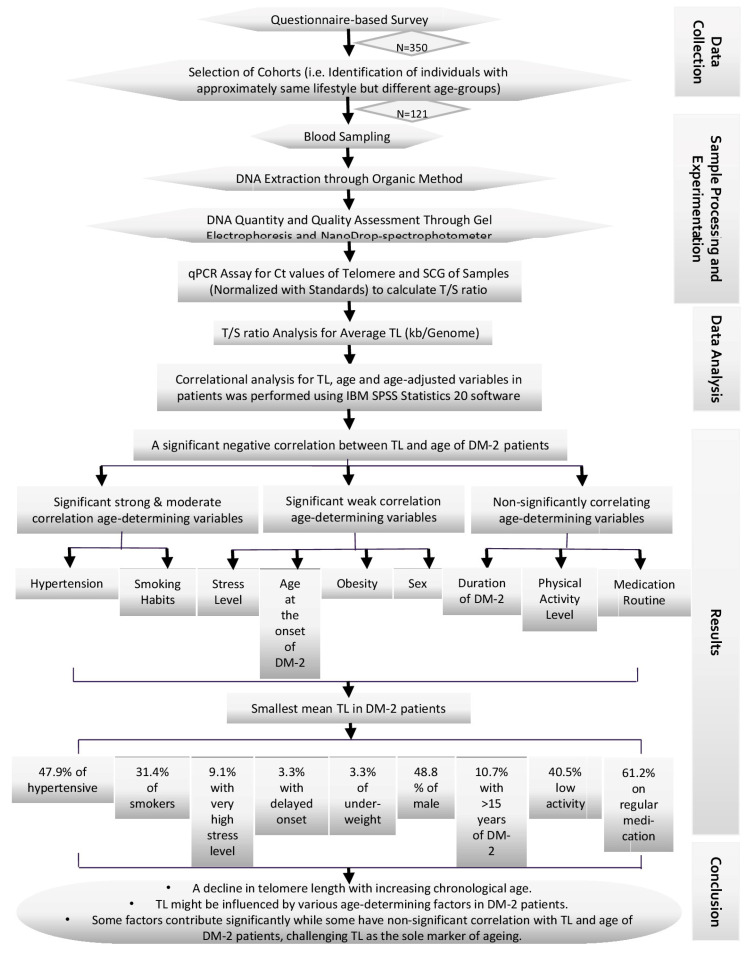
Schematic flowchart of the study.

After calculating the telomere length for each sample, the effects of different variables (sex, smoking, physical activity, stress level, etc.) on telomere length dynamics in diabetic patients were assessed (Figure 2) using IBM SPSS Statistics 20.

## 3. Results

**Figure 3:**
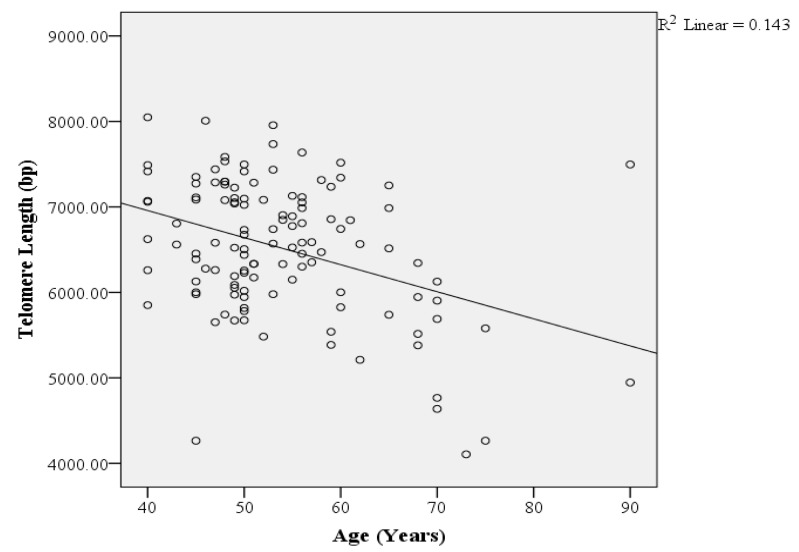
Correlation between telomere length and the age of diabetic patients. A significant moderate negative correlation is presented with a linear regression line.

In this study, there is a significant negative correlation (Figure 3) between increasing age and telomere length (because R^2^ = 0.143, *p*-value = 0.00) in diabetic patients.

Along with the significant correlation of increasing age, the correlations of many other age-determining factors (Table 2) with telomere length, including obesity (based on BMI), onset of diabetes, smoking habits, physical activity rate, hypertension, stress level, sex, and medication, were studied in this research.

**Figure 4:**
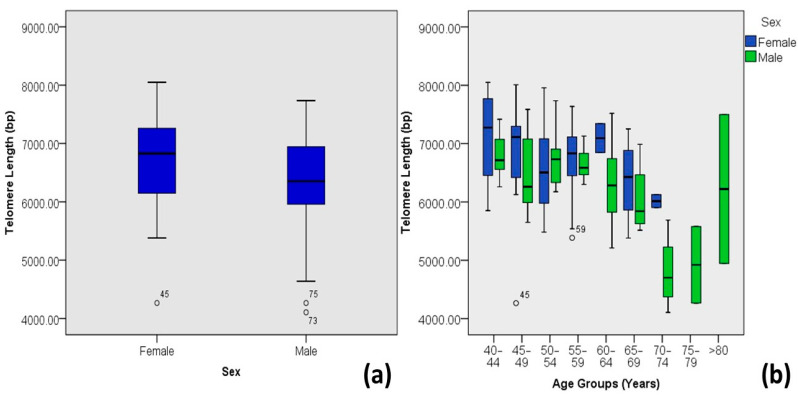
Correlations between sex and age-adjusted telomere length (Outliers are marked by the age of the patients). (**a**) Box plot showing correlations among DM-2 patients and (**b**) plot of age-adjusted correlations.

A typical data set from leukocytes of 59 males and 62 females in the Pakistani population with type 2 diabetes is shown in Table 2. For the females, the leukocytes had a mean telomere length of 6.68 kb/diploid genome (with the longest telomere length in the age group of 40–44-year-old individuals), while in males, leukocytes had a mean TL of 6.35 kb/diploid genome (with the longest telomere length in the age group of 50–54-year-old individuals), as shown in Figure 4.

**Figure 5:**
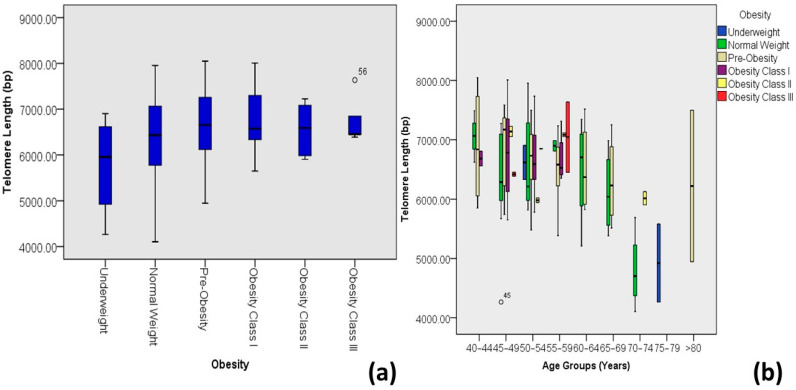
Correlations between obesity and age-adjusted telomere length (Outliers are marked by the age of the patients). (**a**) Box plot showing correlations among DM-2 patients and (**b**) plot of age-adjusted correlations.

For obesity (Table 2), the shortest mean telomere length was observed among underweight diabetic patients. The median telomere length tended to increase with increasing BMI (until pre-obesity), while it again declines as the BMI continues to increase (as in obesity class III), as shown in Figure 5.

**Figure 6:**
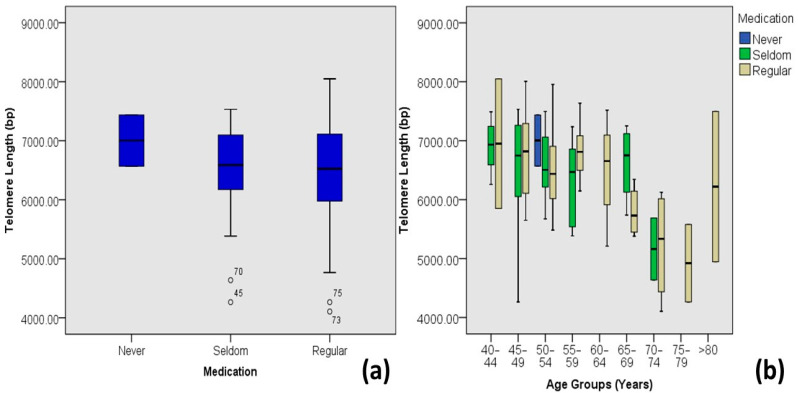
Correlations between routine medication and age-adjusted telomere length (Outliers are marked by the age of the patients). (**a**) Box plot showing correlations among DM-2 patients and (**b**) plot of age-adjusted correlations.

Individuals who have never taken medicine for diabetes tend to have longer telomere lengths as compared to those who seldom or regularly take medicines (Figure 6). However, the individuals with the smallest and the longest telomere lengths were on regular medication.

**Figure 7:**
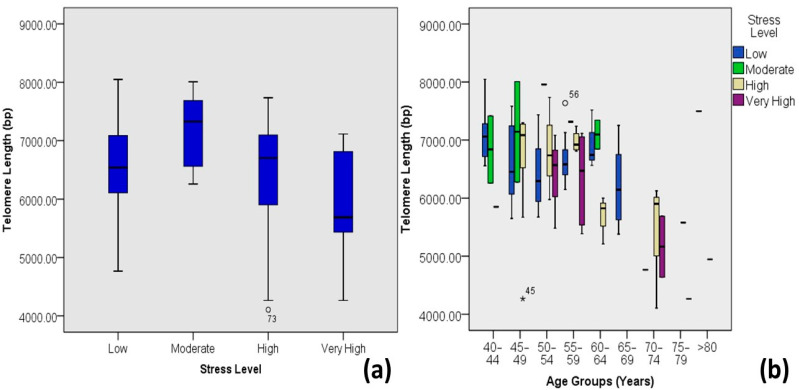
Correlations between stress levels and age-adjusted telomere length (Outliers are marked by the age of the patients). (**a**) Box plot showing correlations among DM-2 patients and (**b**) plot of age-adjusted correlations.

**Figure 8:**
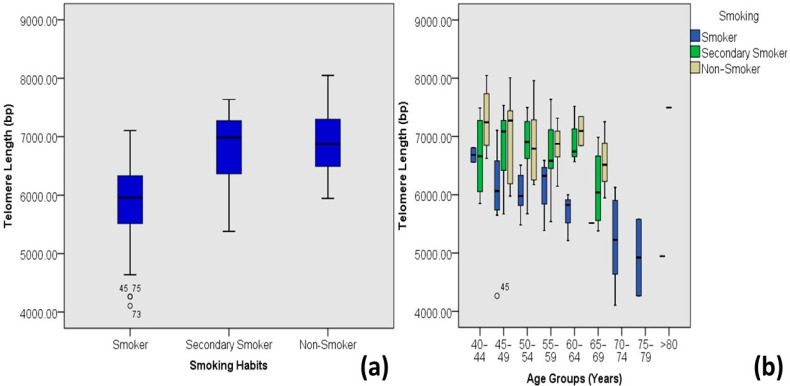
Correlations between smoking habits and age-adjusted telomere length (Outliers are marked by the age of the patients). (**a**) Box plot showing correlations among DM-2 patients and (**b**) plot of age-adjusted correlations.

Diabetic individuals with moderate stress levels tend to have longer mean telomeres compared to individuals with high and very high stress levels (Figure 7). On average, primary smokers tend to have smaller telomere lengths compared to secondary smokers and non-smokers (Figure 8).

**Figure 9:**
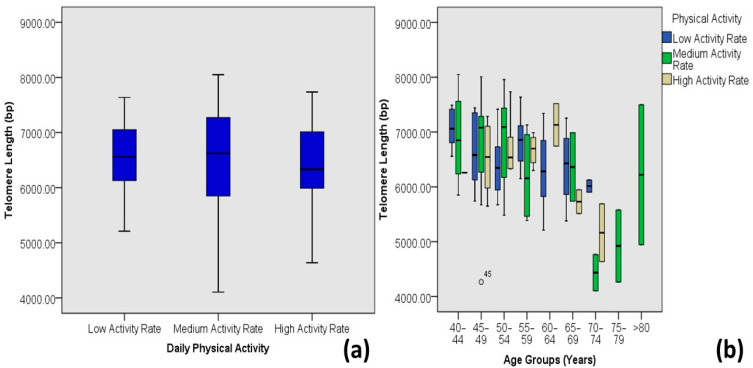
Correlations between physical activity levels and age-adjusted telomere length (Outlier is marked by the age of the patients). (**a**) Box plot showing correlations among DM-2 patients and (**b**) plot of age-adjusted correlations.

Individuals with moderate to higher physical activity have longer mean telomere lengths than individuals with a lower activity rate. On the contrary, the individual with the longest telomere length lies in the cohort of individuals with medium physical activity (Figure 9).

**Figure 10:**
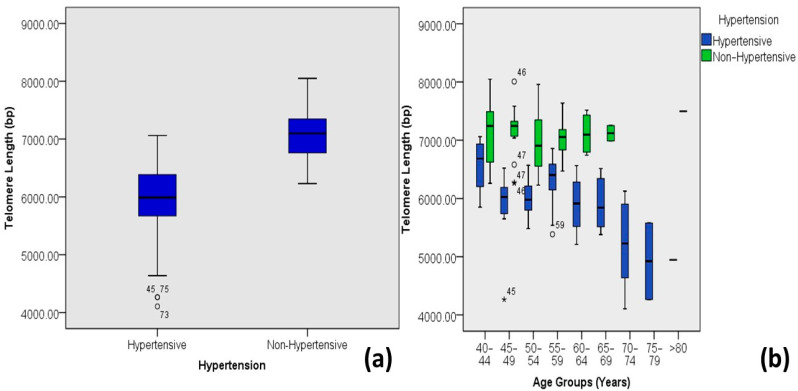
Correlations between hypertension and age-adjusted telomere length (Outliers are marked by the age of the patients). (**a**) Box plot showing correlations among DM-2 patients and (**b**) plot of age-adjusted correlations.

**Figure 11:**
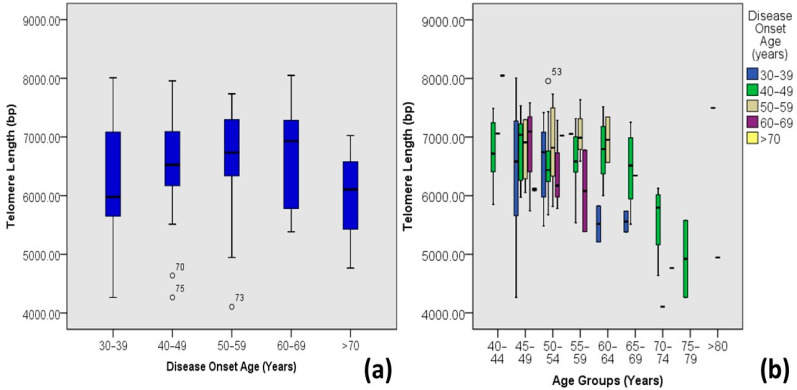
Correlations between the onset of diabetes and age-adjusted telomere length (Outliers are marked by the age of the patients). (**a**) Box plot showing correlations among DM-2 patients and (**b**) plot of age-adjusted correlations.

**Figure 12:**
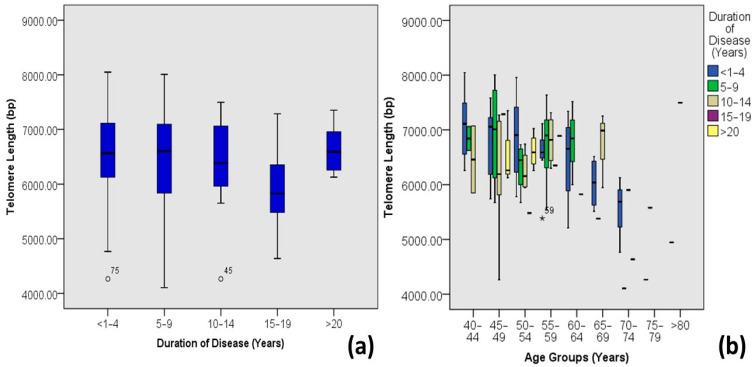
Correlations between the duration of diabetes and age-adjusted telomere length (Outliers are marked by the age of the patients). (**a**) Box plot showing correlations among DM-2 patients and (**b**) plot of age-adjusted correlations.

There was a clear difference in the range (minimum and maximum telomere lengths) and the mean telomere lengths of hypertensive and non-hypertensive individuals with DM-2. Hypertensive diabetic individuals presented smaller telomere lengths than the non-hypertensive individuals (Figure 10). Most of the individuals with earlier onset of DM-2 tends to have shorter telomere length (Figure 11); however, according to this study, the longer the duration of diabetes, the shorter the telomere length on average (Figure 12).

## 4. Discussion

Telomere length assays help us to understand the mechanisms of aging because telomere length serves as a unique cellular and molecular marker for studying the aging cell. Many variables are known to influence the telomere length in diabetic patients, including sex, diabetes type, geographical region, BMI, parental age, exercise, oxidative stress, genotype, smoking status, inherited mutations for accelerated aging syndrome, and psychological stress [19]. Although this study presents a few limitations, including the comparatively small sample size in terms of the number of patients (121 patients selected out of 350 patients from the questionnaire-based analysis), the strength of this study lies in the fact that the samples were collected from a homogenous population to avoid the maximum effect of extraneous variables, including geographical characteristics, patient’s medical history, use of supplements and dietary habits, sleep time, exhaustive physical activity, tobacco consumption other than smoking, lifestyle variations, age, fertility, and marital status. Therefore, even if additional research with a large sample size will be required to support the results of this study, the sound sampling strategy is the strength of this research, exposing a phenomenon that merits in-depth analysis.

Not only the aging mechanism but also several studies have supported the theory of the association of telomere length with age-related diseases, cancer, and lifestyle changes (e.g., smoking and physical activity). Extensive epidemiological studies on different populations are used to explain or deny the correlations between telomere length and a range of diseases [19]. Many diseases are found to be correlated with a shorter telomere length, including ischemic heart disease [23], lung cancer among smokers [24], Alzheimer’s disease [25], high blood pressure [26], diabetes [27], aging [28], and dementia [29]; many of the studies indicate that a few diseases are not significantly correlated with the telomere length, including colorectal cancer [30], age-related macular degeneration [23], and death from infections, cardiac or cerebrovascular disease, or cancer [10].

Diabetes is a serious, chronic condition affecting the lives of individuals and populations worldwide. In 2017, it was projected to have caused four million deaths worldwide, ranking among the top 10 causes of mortality for people [31]. As of 2019, the global prevalence of diabetes was estimated to be 9.3% (about 463 million adults aged 20–79 years). China, India, and Pakistan are expected to have the highest number of diabetic individuals in 2045, with counts of 147, 134, and 37 million, respectively [32]. 

Different researchers have indicated correlations between a shorter leukocyte telomere length and diabetes and diabetes-associated complications, including impaired glucose tolerance and diabetic macroangiopathy [19]. However, it is yet unknown how diabetes affects TL and molecular age. While some researchers [33,34] have found no age-related telomere length attrition between patients with diabetes mellitus and non-diabetic individuals, others [35,36] have shown an age effect. An association of a shorter telomere length, aging, and diabetes can be alarming for a developing country (Pakistan), which had an increase of 62% in diabetes cases (of 20–79 years individuals) in the past ten years [32]. However, this research indicated a significant moderate negative correlation (Figure 3) between telomere length and the age of diabetic patients, but the mean telomere length tended to decline with an increased duration of diabetes (Figure 12), indicating that although the age of diabetic patients directly influences the telomere length, a longer period of the disease will result in a shorter telomere length. It can be hypothesized that patients with extended periods of DM-2 are exposed to more oxidative stress and related complications of the disease as the chronological age of the patient increases.

The correlation between telomere length and the age of diabetic patients, however, might be accompanied by several other age-determining factors highlighted in this study. Organ dysfunction brought on by aging and diabetes is caused by comparable molecular pathways. The abundance of senescent cells in various tissues increases with age, obesity, and diabetes [37]. Although obesity is associated with shorter telomeres overall [38], studies of older individuals found no relation between telomere length and obesity and no relation with mortality [39]. The present study also indicates a significant but weak correlation (R^2^ = 0.04) between obesity and telomere length variations among diabetic patients; however, it can be noticed (Figure 5) that the median telomere length increases as the BMI increases but before the obesity limit, i.e., pre-obese individuals; once the BMI crosses the obesity threshold, the median telomere length declines in diabetic individuals.

Studies indicate the association between physical activity levels and telomere length dynamics. Edwards and Loprinzi [40] and Stenbäck et al. [41] independently reported a significant relationship between the physical activity level and telomere length, such that moderate physical activity levels are correlated with a significantly longer peripheral blood mononuclear cell TLs compared to the highest and the lowest quartiles of physical activity. The present study also indicates a weak correlation (R^2^ = 0.002) between telomere length and the physical activity levels of diabetic patients. However, individuals with moderate physical activity rates tend to have longer telomere lengths on average compared to those with lower or higher activity rates.

Several papers have reported that hypertensive individuals are associated with a shorter telomere length [42,43,44]. This study also indicates that hypertensive individuals tend to have shorter telomere lengths than non-hypertensive patients, with a significant strong correlation (R = 0.725 and *p* < 0.001).

Stressful lifestyles caused by hectic work schedules, family issues, financial burdens, or emotional damage have been correlated with telomere length variations. Many studies suggest that telomere attrition caused by stress could lead to premature aging without adequate recovery [45]. The present study also indicates a significant negative correlation (Pearson’s correlation coefficient = −0.191, *p*-value < 0.05) between telomere length and stress levels in diabetic patients, such that individuals with high or very high stress levels have shorter mean telomere lengths than those having low or moderate stress levels.

The prevention of TL attrition is also associated with healthy life choices, such as not smoking tobacco and drugs or medicines [24,46]. In this study, a highly significant moderate correlation (R = 0.547 and *p* value = 0.000) is observed between telomere length and smoking habits, and a non-significant negligible correlation (Pearson’s coefficient = −0.05, *p* value = 0.576) with regular medication of diabetic individuals. Smokers tend to have shorter telomere lengths as compared to secondary smokers or non-smokers, and individuals who never had taken medicines for diabetes have longer average telomere lengths (7.0 kb) than those who take medicines seldom (6.54 kb) or regularly (6.50 kb).

The clinical and medical applications of the telomere length analysis include prognostic markers and diagnostic indicators [47], cancer research and treatment [48], implications and evaluations of anti-aging therapies [49], and many more. The current study can facilitate future implications to identify factors that have a range of influence (from strong to weak and from significant to non-significant) on the age-adjusted telomere length in DM-2 patients, thus evaluating the premature aging process and its recovery.

## 5. Conclusions

RT-qPCR is a powerful tool for telomere assays but detects average telomere length, but not on an individual chromosome basis. This method is also extremely sensitive, and so cross-contamination was avoided and the method of analysis was planned appropriately to nullify the chances of erroneous or false results.

The study was conducted on DM-2 patients from Pakistan. The statistical measure of this study suggests a significant negative linear relation between the age of individuals with DM-2 and their telomere length. This finding is important because telomere length is often considered a potential marker of the aging process. However, other factors beyond chronological age may also be influential in determining telomere length, particularly in type 2 diabetes mellitus patients.

Through this research, it is concluded that telomere length may not be the only marker for the aging mechanism as many of the other age-determining factors are worth noting to also contribute to the aging process with significant moderate to strong correlations with telomere length (e.g., smoking habits and hypertension) and significant but weak correlations with telomere length (e.g., sex, obesity, stress level, and age at the onset of diabetes), while some have a non-significant correlation with telomere length (e.g., routine medication, rate of physical activity, and the duration of diabetes) in DM-2 patients. So, this study paved the way to determine the underlying factors that can be further studied to lower the effect of aging or pre-mature aging on diabetic patients.

One more breakthrough of this research includes the further analysis of de-trending cases (individuals with longer telomeres even at older ages and individuals with the shortest telomeres even at younger ages) by asking them for their complete medical checkups and through insights based on their lifestyle. The person with a shorter telomere length was found to be suffering from invasive stage II breast cancer, while the individual with a longer telomere length never had taken any allopathic medicine in his whole life span. So, if properly organized, this assay method (telomere length analysis using qPCR) can also be used as a preliminary diagnostic method for diseases like cancer.

## 6. Recommendations

Real-time qPCR is an efficient method to measure telomere length, even for large sample sizes requiring a minimal amount of DNA, not only to investigate the correlation with age-related diseases but also for diagnostic purposes to find pre-mature aging in individuals and for other diseases like cancer so that such disorders can be timely treated or cured.

There is a need for further study to find mechanisms of cellular aging, senescence, and disease states where this assay method fails to show a significant correlation of telomere length with age-related factors and diseases. Such studies will help to shift the focus of future aging research from disease-oriented studies to cellular- or molecule-oriented studies.

Although this study finds a correlation between the onset of diabetes and telomere shortening, leading to the theory that diabetes accelerates the molecular aging process, there is a need for further research to fully understand the underlying mechanisms and the extent of the impact of diabetes on telomere length dynamics.

## Figures and Tables

**Table 1:** Serial dilutions of standards.

Telomere Standard (ng/µL)	SCG Standard (ng/µL)
6.10	1.8
3.63	1.07
2.16	0.64
1.29	0.38
0.77	0.23

**Table 2:** Age-adjusted telomere length by different age-determining variables in type 2 diabetes patients in Pakistan.

Age Determinants	Variables	Mean Telomere Length (kb)	Minimum and Maximum Lengths (kb)	No. of Patients (N)	%N	*p* Value	R	R^2^
Sex	Male	6.35 ± 0.82	4.11–7.73	59	48.8	0.02	−0.207 *	0.043
	Female	6.68 ± 0.74	4.26–8.05	62	51.2			
Obesity ***	Underweight	5.77 ± 1.14	4.26–6.90	4	3.3	0.025	0.203 *	0.041
	Normal weight	6.31 ± 0.93	4.11–7.96	36	29.8			
	Pre-obesity	6.63 ± 0.72	4.95–8.05	48	39.7			
	Obesity class I	6.72 ± 0.62	5.65–8.01	20	16.5			
	Obesity class II	6.55 ± 0.60	5.90–7.23	8	6.6			
	Obesity class III	6.76 ± 0.53	6.39–7.64	5	4.1			
Medication	Never	7.00 ± 0.61	6.57–7.44	2	1.7	0.576	−0.051	0.003
	Seldom	6.54 ± 0.75	4.26–7.53	45	37.2			
	Regular	6.50 ± 0.79	4.11–8.05	74	61.2			
Stress Level	Low	6.58 ± 0.65	4.77–8.05	68	56.2	0.036	−0.191 *	0.037
	Moderate	7.18 ± 0.67	6.26–8.01	8	6.6			
	High	6.44 ± 0.89	4.11–7.73	34	28.1			
	Very High	5.94 ± 0.99	4.26–7.11	11	9.1			
Smoking Habits	Primary Smokers	5.83 ± 0.73	4.11–7.10	38	31.4	0.000	0.547 **	0.299
	Secondary Smokers	6.77 ± 0.62	5.38–7.64	43	35.5			
	Non-Smokers	6.91 ± 0.57	5.95–8.05	40	33.1			
Physical Activity	Low Activity	6.47 ± 0.69	5.21–7.64	49	40.5	0.587	−0.050	0.002
	Medium Activity	6.59 ± 0.88	4.11–8.05	45	37.2			
	High Activity	6.53 ± 0.74	4.64–7.73	27	22.3			
Hypertension	Hypertensive	5.93 ± 0.64	4.11–7.06	58	47.9	0.000	0.725 **	0.526
	Non-Hypertensive	7.07 ± 0.44	6.23–8.05	63	52.1			
Disease Onset Age	30–39	6.27 ± 0.99	4.26–8.01	17	14.0	0.005	−0.254 **	0.065
	40–49	6.56 ± 0.68	4.26–7.96	71	58.7			
	50–59	6.62 ± 0.92	4.11–7.73	19	15.7			
	60–69	6.7 ± 0.88	5.39–8.05	10	8.3			
	>70	6.00 ± 0.93	4.77–7.02	4	3.3			
Duration of Diabetes	<1–4	6.59 ± 0.79	4.26–8.05	61	50.4	0.416	−0.075	0.006
	5–9	6.50 ± 0.88	4.11–8.01	24	19.8			
	10–14	6.46 ± 0.76	4.26–7.5	23	19.0			
	15–19	5.92 ± 0.99	4.64–7.29	5	4.1			
	>20	6.64 ± 0.43	6.13–7.35	8	6.6			

* The correlation is significant at the 0.05 level (2-tailed). ** The correlation is significant at the 0.01 level (2-tailed). *** According to the WHO standards (2010). The arithmetic mean (95% CI) was used to calculate the corresponding telomere length in kb, and linear correlation was used to find the significant value (2-tailed), Pearson’s correlation coefficient (R), and R-square values.

## Data Availability

The authors can furnish supporting data and datasets for this research via email upon request.

## Supplementary Materials

The following supporting information can be downloaded at https://www.mdpi.com/article/10.3390/medicina60050698/s1. Supplemental Table S1: Oligomers (Standards and Primers) used for qPCR analysis of Telomere Length; Supplemental Table S2: Reaction Mix for 20 μL of Final Volume.

## Abbreviations

Body mass index (BMI); cycle threshold value (Ct value); diabetes mellitus type 2 (DM-2); ethylene diamine tetra acetic acid (EDTA); kilobase pair (kb); quantitative real-time polymerase chain reaction (qRT-PCR); single copy gene (SCG); telomere length (TL).
