# Supplementary material for: Effects of Age and Biological Age-Determining Factors on Telomere Length in Type 2 Diabetes Mellitus Patients
Source: Medicina (Kaunas). 2024 Apr 24;60(5):698. doi: 10.3390/medicina60050698 (PMC11122877; doi:10.3390/medicina60050698)
Supplement: Supplementary file 1 [file medicina-60-00698-s001.zip › medicina-2969616-supplementary.pdf]

Supplemental Table S1: Oligomers (Standards and Primers) used for qPCR analysis of Telomere Length

Oligomer Type	Oligomers	Sequence (5'-3')	Amplicon Size
Standards	Telomere Standard	TTAGGGTTAGGGTTAGGGTTAGGGTTAGGGT TAGGGTTAGGGTTAGGGTTAGGGTTAGGGTT AGGGTTAGGGTTAGGGTTAGGG	84 bp
	β-Globin/ SCG Standard	GCTTCTGACACAACTGTGTTCACTAGCAACC TCAAACAGACACCATGGTGCATCTGACTCCT GAGGAGAAGTCTGCCGTTACTGCCCTGTGGG GCAAGGTGAACGTGGATGAAGTTGGTG	120 bp
Primers	Telo-F	CGGTTTGTTTGGGTTTGGGTTTGGGTTTGGG TTTGGGTT	>76 bp
	Telo-R	GGCTTGCCTTACCCTTACCCTTACCC TTACCCTTACCCT	
	SCG-F	GCTTCTGACACAACTGTGTTCACTAGC	82 bp
	SCG-R	CACCAACTTCATCCACGTTCACC	

Supplemental Table S2: Reaction Mix for 20 µl of Final Volume

TELOMERE			SCG		
**Components**	**Volumes**	**Final Conc.**	**Components**	**Volumes**	**Final Conc.**

Master mix	10 µl	1X	Master mix	10 µl	1X
DNA or serial dilution	2 µl	10 ng	DNA or serial dilution	2 µl	10 ng
Telo F	0.75 µl		SCG F	0..75 µl	
Telo R	1.25 µl		SCG R	1.25 µl	
H_20	6 µl		H_20	6 µl	
**Final Volume**	20 µl			20 µl	
